# Disparities in the surgical staging of high-grade endometrial cancer in the United States

**DOI:** 10.1186/s40661-016-0036-3

**Published:** 2017-01-19

**Authors:** Jonathan R. Foote, Stephanie Gaillard, Gloria Broadwater, Julie A. Sosa, Brittany Davidson, Mohamed A. Adam, Angeles Alvarez Secord, Monica B. Jones, Junzo Chino, Laura J. Havrilesky

**Affiliations:** 1 Division of Gynecologic Oncology, Duke University Medical Center, 2301 Erwin Rd, Durham, NC 27710 USA; 2 Biostatistics, Duke Cancer Institute, Duke University, 2301 Erwin Rd, Durham, NC 27710 USA; 3 Department of Surgery, Duke University Medical Center, 2301 Erwin Rd, Durham, NC 27710 USA; 4 Duke Clinical Research Institute, Durham, USA; 5 Division of Radiation Oncology, Duke University Medical Center, 2301 Erwin Rd, Durham, NC 27710 USA

**Keywords:** Endometrial cancer, Disparity, High-grade, Staging, NCDB, Surgical volume

## Abstract

**Background:**

The National Comprehensive Cancer Network (NCCN) and the Society of Gynecologic Oncology (SGO) recommend lymph node sampling (LNS) as a key component in the surgical staging of high-grade endometrial cancer. Our goal was to examine surgical staging patterns for high-grade endometrial cancer in the United States.

**Methods:**

The National Cancer Data Base (NCDB) was searched for patients who underwent surgery for serous, clear cell, or grade 3 endometrioid endometrial cancer. Outcomes were receipt of LNS and overall survival (OS). Multivariate logistic regression was used to examine receipt of LNS in Stage I–III disease based on race (White vs. Black), income, surgical volume, and distance traveled to care. Multivariate Cox proportional hazards regression modeling was used to assess OS based on stage, race, income, LNS, surgical volume, and distance traveled.

**Results:**

Forty-two thousand nine hundred seventy-three patients were identified: 76% White, 53% insured by Medicare/Medicaid, 24% traveled >30 miles, and 33% stage III disease. LNS was similar among White and Black women (81% vs 82%). LNS was more common among >30 miles traveled (84% vs 81%, *p <* 0.001), higher surgical volume (83% vs 80%, *p <* 0.001), and academic centers (84% vs 80%, *p <* 0.001). In multivariate analysis, higher income, higher surgical volume, Charlson-Deyo score, and distance traveled were predictors of LNS. Stage III disease (HR 3.39, 95% CI 3.28–3.50), age (10-year increase; HR 1.63, 95% CI 1.61–1.66), lack of LNS (HR 1.64, 95% CI 1.56–1.69), and low income (HR 1.20, 95% CI 1.14–1.27) were predictors of lower survival.

**Conclusions:**

Surgical care for high-grade endometrial cancer in the United States is not uniform. Improved access to high quality care at high volume centers is needed to improve rates of recommended LNS.

## Background

Endometrial cancer is the most common form of gynecologic cancer in the United States, with more than 60,000 new cases and 10,470 deaths estimated to occur in 2016 [1]. The majority of endometrial cancers are Type I, grade 1–2 endometrioid adenocarcinoma and have an excellent prognosis, most often presenting as low-grade and at an early stage. Conversely, Type II cancers, including uterine papillary serous carcinoma (UPSC), clear cell carcinoma (CC), and grade 3 endometrioid adenocarcinoma, often have a much worse prognosis, presenting with extra-uterine disease [2–5]. UPSC, CC, and grade 3 endometrioid adenocarcinoma represent 10–27% of incident endometrial cancer cases, but account for over 70% of endometrial cancer deaths [6].

The National Comprehensive Cancer Network (NCCN) and the Society of Gynecologic Oncology (SGO) support pelvic and para-aortic lymphadenectomy for patients with high-grade endometrial cancer, including UPSC, CC, and grade 3 endometrioid adenocarcinoma [7–9]. Although the surgical guidelines are clear, there is a demonstrated racial disparity between Whites and Blacks in de facto surgical management [10]. Black women are less likely to undergo surgery for endometrial cancer [11–13]. While many authors have examined associations between race and the epidemiology, treatment, and outcomes of endometrial cancer, no author has explored the relationship between race and the type of surgery performed. Disparities also exist for the distance traveled to receive care for endometrial cancer, although the available data are limited. In 2011, Benjamin et al. performed a statewide analysis of endometrial cancer care in Arizona and found that minorities travel farther than Whites to receive care, while patients with government-funded insurance travel farther than patients with other types of insurance [14]. There are no other regional or national studies examining the relationship between receipt of care for endometrial cancer and travel distance. Based on the NCCN and the SGO recommendations of lymph node sampling during the surgical staging of high-grade endometrial cancer, we explored surgical practice patterns and disparities in the treatment of UPSC, CC, and grade 3 endometrioid adenocarcinoma in the United States.

## Methods

### Data source

The National Cancer Data Base (NCDB) is a national oncology outcomes database in the United States, founded in 1989 by the American College of Surgeons (ACS) and its Commission on Cancer (CoC) Program. One of the largest cancer registries in the world, the NCDB collects data on 70% of all new cancer diagnoses in the U.S. from over 1,500 reporting hospitals [15]. The NCDB data coding process is standardized according to the CoC Registry Operations and Data Standards Manual, the American Joint Committee for Cancer (AJCC) Manual for Staging of Cancer, and the International Classification of Disease for Oncology, Third Edition (ICD-O3). For the current study, all data were extracted from medical records by trained and certified tumor registrars. Our Institutional Review Board granted this study an exemption status due to the de-identified nature of the dataset.

### Case selection

The NCDB Participant User File was searched for all patients who underwent surgery for UPSC, CC, or grade 3 endometrioid adenocarcinoma. Data were available from the years 1998 to 2012. Demographic and socioeconomic information was recorded for each patient, including age, race, income, education and insurance status. Clinical and pathologic data collected included International Federation of Gynecology and Obstetrics (FIGO) staging, Charlson-Deyo comorbidity score, number of regional lymph nodes examined, number of para-aortic lymph nodes examined, peritoneal cytology status, and performance of omentectomy. While FIGO staging criteria were updated in 2009, we did not reclassify patients for our analysis. The FIGO 2009 staging criteria changes to Stage I would not alter our Stage I designation; and changes to Stage II and Stage III designation could not be fully addressed from data available in the NCDB. Patients were further categorized based on treatment center type (academic research center, community cancer program/other, comprehensive community cancer program), hospital endometrial cancer surgical volume, and distance travelled to receive care (≤ or > 30 miles). Surgical volume was defined based on strategies previously utilized in NCDB-related publications [16]. Hospital surgical volume was annualized based on the total number of endometrial cancer patients and the total number of years of reported data. Surgical volume groups were then categorized by quartile rank: 75^th^ percentile = very-high volume (>31.7 cases/year); 51^st^–75^th^ percentile = moderately-high volume (13.3–31.6 cases/year); 25^th^–50^th^ percentile = intermediate volume (6.7–13.2 cases/year); and <25^th^ percentile = low volume (<6.7 cases/year). While distance to care was recorded continuously in the NCDB, we stratified distance in two groups, ≤ 30 miles and > 30 miles, based on the overall mean distance traveled (mean *=* 30).

### Statistical analysis

The main outcomes examined were the rates of lymph node sampling and overall survival (OS). Multivariate logistic regression was used to explore differences in receipt of lymph node sampling (LNS). Stage IV patients were excluded as LNS may not always be necessary or indicated in this patient population. Given that there is not a standard number of sampled lymph nodes to constitute ‘adequate’ staging, we performed a sensitivity analysis using replicate logistic models considering various cut points for the number of lymph nodes sampled: (1) 0 vs. 1+; (2) 0–1 vs. 2+; (3) 0–4 vs. 5+; (4) 0–9 vs. 10+; (5) 0–14 vs. 15+; (6) 0–19 vs. 20+. In addition, logistic regression modeling was used to explore a subset of patients with available data on para-aortic LNS. The following variables were assessed for possible associations with receipt of LNS (as categorized by the NCDB): race (White, Black, other), median-household income (defined based on a specific patient’s zip code at the time of diagnosis compared against 2012 American Community Survey data: <$38,000; $38,000 to $47,999; $48,000 to $62,999; or > $63,000), education (defined as the percentage of the population with a high school diploma as the highest degree obtained in a specific patient’s zip code: ≥21%, 13–20%, 7–12.9%, or <7%), insurance status (not insured, private insurance, Medicaid, Medicare, or other), Charlson-Deyo comorbidity score (Charlson score of 0, 1, or ≥2), type of treatment center (comprehensive community cancer program, community cancer program/other, or academic research center), hospital endometrial cancer surgical volume, and distance traveled to receive care. Receipt of peritoneal cytology and omentectomy were inconsistently reported, and therefore excluded from our assessment. Given limited numbers regarding minorities other than Blacks, we grouped additional minorities as ‘other’ for model parsimony. Table 1 lists patient and clinical characteristics based on race for patients with Stage I–III disease. Categorical variables were compared using chi-square tests. Multivariate Cox proportional hazards regression modeling and Kaplan Meier curves were used to assess associations between clinical variables and OS. OS models were explored utilizing distance to care as both continuous and dichotomous (≤30 miles and > 30 miles). Statistical significance was considered a priori at a two-sided *p*-value of <0.05. We defined a clinically significant difference between categorical groups to exist if a *p*-value of <0.05 was associated with a ≥5% difference. Statistical analyses were conducted using SAS v. 9.3 software (SAS Institute, Inc., Cary, NC) and survival plots were created using Spotfire S+ v. 8.1 (TIBCO, Palo Alto, CA).

**Table 1:** Patient characteristics of patients with UPSC, CC, or grade 3 endometrioid carcinoma

	Overall	Black	White	Other	*p*-values
	n (%)	n (%)	n (%)	n (%)	
Total	42,973 (100)^a^	5,285 (12.2)	35,694 (76.1)	1,417 (3.3)	
Age at diagnosis					
*Mean (SD)*	66.0 (11.5)	65.7 (11.7)	66.2 (11.7)	61.5 (11.6)	<0.001
Income					
*< $38,000*	7,708 (17.9)	2,352 (44.5)	5,119 (14.3)	167 (11.8)	<0.001
*$38,000–$47,999*	9,814 (22.9)	1,175 (22.2)	8,304 (23.3)	216 (15.2)	
*$48,000–$62,999*	11,218 (26.1)	921 (17.4)	9,747 (27.3)	375 (26.5)	
*≥ $63,000*	13,192 (30.7)	707 (13.4)	11,668 (32.7)	627 (44.2)	
*Unknown*	1,041 (2.4)				
Insurance status					
*Uninsured*	1,418 (3.3)	256 (4.8)	1,060 (3.0)	81 (5.7)	<0.001
*Private Insurance*	17,582 (40.9)	1,807 (34.2)	14,845 (40.6)	683 (48.2)	
*Medicaid and Medicare*	22,750 (52.9)	3,081 (58.3)	18,823 (52.7)	578 (40.8)	
*Other*	282 (0.7)	30 (0.6)	218 (0.6)	33 (2.3)	
*Unknown*	941 (2.2)				
Education^b^					
*≥ 21%*	7,196 (16.7)	1,928 (36.5)	4,881 (13.7)	318 (22.4)	<0.001
*13–20%*	10,792 (25.1)	1,923 (36.4)	8,393 (23.5)	344 (24.3)	
*7.0–12.9%*	13,867 (32.3)	933 (17.7)	12,314 (34.5)	413 (29.1)	
*< 7.0%*	10,107 (23.5)	373 (7.1)	9,277 (26.0)	310 (21.9)	
*Unknown*	1,011 (2.4)				
Charlson-Deyo Score					
*0*	24,653 (57.4)	2,858 (54.1)	20,540 (57.5)	890 (62.8)	<0.001
*≥ 1*	8,128 (18.9)	1,307 (24.7)	6,465 (18.1)	258 (18.2)	
*Unknown*	10,192 (23.7)				
Histology					
*Uterine papillary Serous*	8,238 (19.1)	1,506 (28.5)	6,413 (18.0)	211 (14.9)	<0.001
*Clear cell*	3,631 (8.5)	576 (10.9)	2,873 (8.0)	134 (9.5)	
*Grade 3 endometrioid*	31,104 (72.4)	3,203 (60.6)	26,408 (74.0)	1,072 (75.6)	
FIGO Stage					
*I*	24,052 (55.9)	2,622 (49.6)	20,348 (57.0)	756 (53.4)	<0.001
*II*	4,888 (11.4)	764 (14.5)	3,911 (11.0)	152 (10.7)	
*III*	14,033 (32.7)	1,899 (35.9)	11,435 (32.0)	509 (35.9)	
Lymph node sampling^c^					
*Yes*	35,158 (81.8)	4,265 (80.7)	29,211 (81.8)	1,214 (85.7)	<0.001
*No*	7,815 (18.2)	1,020 (19.3)	6,483 (18.2)	203 (14.3)	
Treatment center type					
*Community Cancer Program*	2,647 (6.2)	269 (5.1)	2,258 (6.3)	108 (7.6)	<0.001
*Comprehensive Community Cancer Program*	22,272 (51.8)	2,225 (42.1)	19,191 (53.8)	621 (43.8)	
*Academic/ Research Program*	18,054 (42.0)	2,791 (52.8)	14,245 (39.9)	688 (48.6)	
Hospital surgical volume^d^					
*Very–high*	30,518 (71.0)	3,880 (73.4)	25,146 (70.5)	1,006 (71.0)	<0.001
*Moderately–high*	7,998 (18.6)	926 (17.5)	6,709 (18.8)	296 (20.9)	
*Intermediate*	3,272 (7.6)	379 (7.2)	2,796 (7.8)	83 (5.9)	
*Low*	1,185 (2.8)	100 (1.9)	1,043 (2.9)	32 (2.2)	
Distance to care					
*≤ 30 miles*	31,645 (73.6)	4,246 (80.3)	25,884 (72.5)	1,152 (81.3)	<0.001
*> 30 miles*	10,366 (24.1)	913 (17.3)	9,025 (25.3)	234 (16.5)	
*Unknown*	962 (2.3)				

^a^Race data is available for 42,396 patients

^b^Defined as the percentage of the population with a high school diploma in a specific patient’s area code

^c^Defined based on the number of regional lymph nodes sampled during surgery. Yes = 1+; No = 0

^d^Surgical volume groups were identified based on quartile rank of the total number of endometrial cancer patients undergoing surgery and the total number of years of reported data: 75^th^ percentile = very-high volume (>31.7cases/year); 51^st^–75^th^ percentile = moderately-high volume (13.3–31.6 cases/year); 25^th^–50^th^ percentile = intermediate volume (6.7–13.2 cases/year); and <25^th^ percentile = low volume (<6.7 cases/year)

## Results

### Patient and cancer characteristics

A total of 53,841 patients were identified who underwent surgery and had available lymph node status data between 1998 and 2012 for high-grade endometrial cancer, including UPSC, CC, and grade 3 endometrioid adenocarcinoma. Patients were excluded from our analysis if lymph node status was not available. After excluding Stage IV patients, 42,973 patients were available for analysis. Table 1 lists demographic, socioeconomic, and clinical characteristics. Grade 3 endometrioid adenocarcinoma accounted for 31,104 cases (72%), while UPSC accounted for 8,238 cases (19%), and CC accounted for 3,631 cases (9%). Sixty–seven percent (*n =* 28,940) had Stage I/II disease, while 33% (*n =* 14,033) had Stage III disease. Seventy–six percent of patients were White (*n =* 35,694), while 12% were Black (*n =* 5,285). The majority of patients were insured via Medicaid and Medicare (53%) or private insurance (41%). Most patients underwent surgery at a comprehensive community cancer program (52%) or at an academic/research center (42%). Seventy–one percent of patients received surgery at very-high volume centers (>31.7 cases/year). The mean distance traveled to care was 30 miles.

### Receipt of surgical staging

In our cohort of 42,973 patients, 35,158 (82%) underwent regional LNS. In univariate analysis, receipt of care at an academic center (84% vs 70%, *p <* 0.001) and at a hospital with higher endometrial cancer surgical volume (>75^th^ percentile, 83% vs 71% (all others), *p <* 0.001) were both associated with receipt of LNS (data not shown).

In multivariate analysis, income, distance traveled to care, Charlson-Deyo comorbidity score, and hospital surgical volume were all significant predictors of receipt of LNS. The sensitivity of these results were examined using replicate logistic models considering various cut points for the number of lymph nodes removed, which produced identical predictors of more complete staging to those reported above for all replicate models (0 vs. 1+; 0–1 vs. 2+; 0–4 vs. 5+; 0–9 vs. 10+; 0–14 vs. 15+; 0–19 vs. 20+). Surgical volume and type of cancer center were strongly associated with each other, and thus were not considered concomitantly in multivariate analysis: Surgical volume was incorporated into the multivariate model. Receipt of LNS was less likely in patients with a Charleson-Deyo comorbidity score of ≥1 (OR 0.73, 95% CI 0.68 to 0.78) (Table 2), or with an annual income ≤ $38,000 (38 k-$47,999: OR 1.08, 95% CI 0.98 to 1.18; $48 k-$62,999: OR 1.20, 95% CI 1.10 to 1.32; >$63 k: OR 1.33, CI 1.21 to 1.46). Compared to very-high volume hospitals (>31.7 cases/year), receipt of LNS was also less likely in patients receiving surgical care at moderately-high volume hospitals (OR 0.65, 95% CI 0.61 to 0.71), intermediate volume (OR 0.51, 95% CI 0.45 to 0.56), or low volume (OR 0.39, 95% CI 0.33 to 0.45). Receipt of LNS was more likely in patients who traveled >30 miles to receive their care (OR 1.12, 95% CI 1.04 to 1.21).

**Table 2:** Multivariate predictors of receipt of lymph node sampling in UPSC, CC, and grade 3 endometrioid carcinoma

Predictors	Odds Ratio (Reference =1)	95% CI	*p*-value
Race			
*White*	Reference		0.21
*Black*	0.97	0.88–1.06	
*Other*	1.15	0.96–1.36	
Income			
< $38,000	Reference		<0.001
$38,000–$47,999	1.08	0.98–1.18	
*$48,000–$62,999*	1.20	1.10–1.32	
*≥ $63,000*	1.33	1.21–1.46	
Surgical Volume^a^			
*Very high*	Reference		<0.001
*Moderately high*	0.65	0.61–0.71	
*Intermediate*	0.51	0.45–0.56	
*Low*	0.39	0.33–0.45	
Charlson-Deyo score			
*0*	Reference		<0.001
*1*	0.73	0.68–0.78	
Distance to care			
*≤ 30 miles*	Reference		0.0025
*> 30 miles*	1.12	1.04–1.21	

^a^Surgical volume groups were identified based on quartile rank: 75^th^ percentile = very-high volume (>31.7cases/year); 51^st^–75^th^ percentile = moderately-high volume (13.3–31.6 cases/year); 25^th^–50^th^ percentile = intermediate volume (6.7–13.2 cases/year); and <25^th^ percentile = low volume (<6.7 cases/year)

In our subset analysis of receipt of para-aortic LNS, there were 11,068 evaluable patients with para-aortic lymph node sampling status recorded. A total of 5,554 (50%) patients underwent para-aortic LNS. Significant predictors of receipt of para-aortic LNS were similar to our main model, including Black race not being associated with a difference in receipt of para-aortic LNS (OR 1.03, 95% CI 0.92 to 1.16) (Table 3).

**Table 3:** Multivariate predictors of receipt of para-aortic lymph node sampling in UPSC, CC, and grade 3 endometrioid carcinoma

Predictors	Odds Ratio (Reference =1)	95% CI	*p*-value
Race			
*White*	Reference		0.79
*Black*	1.03	0.92–1.16	
*Other*	1.05	0.86–1.28	
Income			
< $38,000	Reference		
$38,000–$47,999	1.12	0.99–1.26	<0.001
*$48,000–$62,999*	1.23	1.09–1.38	
*≥ $63,000*	1.34	1.18–1.50	
Surgical Volume^a^			
*Very high*	Reference		<0.001
*Moderately high*	0.79	0.72–0.88	
*Intermediate*	0.72	0.61–0.85	
*Low*	0.57	0.46–0.72	
Charlson-Deyo score			
*0*	Reference		<0.001
*1*	0.69	0.63–0.76	
Distance to care			
*≤ 30 miles*	Reference		0.069
*> 30 miles*	1.09	0.99–1.19	

^a^Surgical volume groups were identified based on quartile rank: 75^th^ percentile = very-high volume (>31.7cases/year); 51^st^–75^th^ percentile = moderately-high volume (13.3–31.6 cases/year); 25^th^–50^th^ percentile = intermediate volume (6.7–13.2 cases/year); and <25^th^ percentile = low volume (<6.7 cases/year)

### Overall survival

**Fig. 1:**
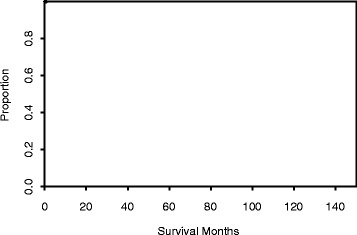
Multivariate predictors of overall survival in high-grade endometrial cancer. **a**. Overall survival curve based on stage of disease. **b**. Overall survival curve based on receipt of lymph node sampling. **c**. Overall survival curve based on race (*White* vs. *Black*). **d**. Overall survival curve based on distance to care (≤30 miles vs. >30 miles)

Multivariate Cox proportional hazards analysis was performed to identify predictors of overall survival while controlling for patient age, race, income, receipt of lymph node staging, stage of disease, adjuvant chemotherapy or radiation, hospital surgical volume, and distance to care (Table 4). A total of 31,647 patients had sufficiently matured data available for survival analysis. Compared to Stage I/II disease, Stage III disease was associated with lower overall survival (HR 3.39, 95% CI 3.28 to 3.50) (Fig. 1a). Patients who received LNS had improved overall survival (HR 0.61, 95% CI 0.59 to 0.64) (Fig. 1b). Although LNS was more common at higher volume surgical centers, surgical volume was not a statistically significant predictor of overall survival (Table 4). Black women compared to White women had a poorer overall survival (HR 1.36, 95% CI 1.29 to 1.42) (Fig. 1c). While patients who traveled >30 miles were more likely to receive LNS, distance to care was not associated with overall survival (HR 1.00, 95% CI 0.99 to 1.00, *p =* 0.758) (Fig. 1d).

**Table 4:** Multivariate predictors of overall survival in UPSC, CC, and grade 3 endometrioid carcinoma

Predictors	Hazard Ratio (Reference = 1)	95% CI	*p*-value
Stage			
*I/II*	Reference		
*III*	3.39	3.28–3.50	<0.001
Age			
*10-year increase*	1.63	1.61–1.66	<0.001
Race			
*White*	Reference		<0.001
*Black*	1.36	1.29–1.42	
*Other*	0.92	0.83–1.02	
Income			
< $38,000	Reference		<0.001
*$38,000–$47,999*	0.96	0.91–1.01	
*$48,000–$62,999*	0.92	0.88–0.97	
*≥ $63,000*	0.83	0.79–0.87	
Lymph node sampling			
*No*	Reference		<0.001
*Yes* ^a^	0.61	0.59–0.64	
Surgical Volume			
*Highest (4* ^*th*^ *) Quartile*	Reference		0.59
*Third Quartile*	0.97	0.94–1.02	
*Second Quartile*	1.02	0.96–1.08	
*Lowest (1* ^*st*^ *) Quartile*	1.01	0.91–1.11	
Distance to care^b^			
*10-mile increase*	1.00	0.99–1.00	0.48

^a^Defined based on the number of regional lymph nodes sampled during surgery. Yes = 1+; No = 0

^b^“Great circle” distance: defined as the distance between the reporting hospital and the patient’s zip code centroid. Defined here continuously. Results were similar when distance was defined as ≤30 miles versus >30 miles

In our multivariate subset analysis of para-aortic LNS, significant predictors of OS were similar to our main model, including Black race (HR 1.27, 95% CI 1.10 to 1.46). Other significant predictors included Stage III/disease (HR 3.14, 95% CI 2.80 to 3.52) and receipt of para-aortic LNS (HR 0.65, 95% CI 0.59 to 0.73). Similar to our main cohort, surgical volume and distance to care were not associated with OS.

## Discussion

The management of high-grade endometrial cancer relies on the cornerstone of surgical staging. The NCCN recommends that surgical staging for high-grade endometrial cancer, including UPSC, CC, and grade 3 endometrioid adenocarcinoma, consist of pelvic and para-aortic lymphadenectomy [7]. This removal of lymph nodes also affords the opportunity to appropriately tailor adjuvant therapy. In this cohort of high-grade endometrial cancer patients, our data, after controlling for the receipt of adjuvant therapy, demonstrate that receipt of regional LNS is associated with improved survival (HR 0.61, 95% CI 0.59 to 0.64). A number of observational studies have also shown that women who undergo lymph node staging have improved clinical outcomes [17–20]. While the interpretation of such observational studies is heavily limited by selection bias, and no randomized trials specific to a high-risk histology cohort have been performed, the removal of lymph nodes not only follows NCCN recommendations for women with UPSC, CC, and grade 3 endometrioid adenocarcinoma, but also guides the use of appropriate adjuvant therapy [7]. Therefore, the lower rates of NCCN-recommended staging of high-grade endometrial cancer at lower surgical volume centers and among lower income women is a point of concern.

Epidemiological studies have demonstrated that Black women, as compared to White women, are disproportionately affected by high-risk histologic types of endometrial cancer and are less likely to undergo surgical management [11–13, 21–24]. Our study is the first to examine factors associated with the receipt of LNS as part of surgical management, and demonstrates that receipt of LNS for high-grade endometrial cancer is similar among Blacks and Whites (81% vs 80%, respectively). While disparities in the surgical management of high-risk endometrial cancer have improved, Black women, compared to White women, have worse overall survival (Table 4) (OR 1.36, 95% CI 1.29 to 1.42).

Prior studies have also demonstrated that women with endometrial cancer are more likely to undergo lymph node dissection at high-volume hospitals compared to low-volume hospitals (66% vs 35%, *p <* 0.001) [25]. However, treatment at high-volume centers has not been shown to improve overall survival [25–27]. Our data support these prior findings with the highest surgical volume centers significantly associated with receipt of LNS, but not resulting in an improved survival. However, lymph node staging remains a key component of surgical staging for endometrial cancer; and allows for tailoring of adjuvant therapies. Adherence to similar treatment guidelines in ovarian cancer has been associated with proximity to care, although similar reports are not available for endometrial cancer [28]. In our analysis, women with high-grade endometrial cancer who traveled farther for their surgical care were more likely to receive LNS (OR 1.12, 95% CI 1.04 to 1.21). Interestingly, 84% of women who traveled >30 miles for care received care at the highest volume centers. Our findings suggest that patients may have traveled farther initially to seek the surgical expertise of higher volume centers, thereby explaining the higher rates of LNS observed among those who traveled farther.

Several limitations to our study are inherent in the use of large clinical and administrative databases. While the Charlson-Deyo score accounts for comorbidities, the NCDB does not record data on body mass index (BMI), which can influence surgical decision making. Additionally, there was inadequate reporting of omentectomy and peritoneal cytology to include in our analysis. Another limitation is interpretation of the number of lymph nodes examined. The NCDB reports the number of lymph nodes examined by a pathologist; however, information distinguishing between pelvic and para-aortic nodal basins is largely incomplete. As sentinel lymph node mapping is being utilized more often in endometrial cancer, the number of lymph nodes needed for adequate surgical staging is likely to decrease while lymph node status becomes even more vital to the management and prognosis of endometrial cancer. In order to broadly examine the surgical staging issue without focusing on nodal counts, we chose to examine lymph node staging as a dichotomous variable.

## Conclusions

Lymph node staging for high-grade endometrial cancer, whether comprehensive lymphadenectomy or sentinel lymph node mapping, is not only recommended by the NCCN and the SGO, but can also assist in the tailoring of adjuvant therapies. While medical comorbidities (as captured here in the Charlson-Deyo score) may provide inherent difficulties to completing surgical staging, the receipt of guideline-based care should not be limited. High-volume centers are best equipped to provide guideline-based care to these high-risk women.

## Abbreviations

ACS: American College of surgeons
AJCC: American joint committee on cancer
BMI: Body mass index
CC: Clear cell
CoC: Commission on cancer
FIGO: International federation of gynecology and obstetrics
LNS: Lymph node sampling
NCCN: National comprehensive cancer network
NCDB: National cancer data base
OS: Overall survival
SGO: Society of gynecologic oncology
UPSC: Uterine papillary serous carcinoma
